# 4-Chloro-*N*′-(3,5-dibromo-2-hy­droxy­benzyl­idene)benzohydrazide

**DOI:** 10.1107/S1600536812009786

**Published:** 2012-03-28

**Authors:** Wei-Guang Zhang

**Affiliations:** a College of Chemistry and Chemical Engineering, Qiqihar University, Qiqihar 161006, People’s Republic of China

## Abstract

The asymmetric unit of the title compound, C_14_H_9_Br_2_ClN_2_O_2_, contains two independent mol­ecules. Both mol­ecules adopt an *E* configuration about the C=N bond. The dihedral angles between the benzene rings are 30.0 (2) and 51.6 (2)° in the two mol­ecules. In the crystal, mol­ecules are linked through N—H⋯O hydrogen bonds, forming chains along the *b* axis. In addition, there is an intra­molecular O—H⋯N hydrogen bond in each mol­ecule.

## Related literature

For the biological properties of hydrazone compounds, see: Ajani *et al.* (2010 ▶); Angelusiu *et al.* (2010 ▶); Zhang *et al.* (2010 ▶); Horiuchi *et al.* (2009 ▶). For the crystal structures of similar hydrazone compounds, see: Ban (2010 ▶); Hussain *et al.* (2010 ▶); Shalash *et al.* (2010 ▶); Khaledi *et al.* (2009 ▶). For related structures reported recently by the author, see: Zhang (2011 ▶, 2012 ▶).
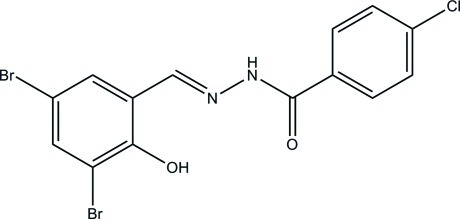


## Experimental

### 

#### Crystal data

C_14_H_9_Br_2_ClN_2_O_2_
*M* *_r_* = 432.50
Monoclinic, 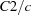

*a* = 21.0503 (19) Å
*b* = 9.9895 (11) Å
*c* = 30.185 (2) Å
β = 101.836 (2)°
*V* = 6212.4 (10) Å^3^
*Z* = 16
Mo *K*α radiation
μ = 5.40 mm^−1^
*T* = 298 K
0.20 × 0.18 × 0.17 mm

#### Data collection

Bruker APEXII CCD area-detector diffractometer
Absorption correction: multi-scan (*SADABS*; Sheldrick, 1996 ▶) *T*_min_ = 0.412, *T*_max_ = 0.461
21727 measured reflections
5775 independent reflections
2828 reflections with *I* > 2σ(*I*)
*R*_int_ = 0.093

#### Refinement

*R*[*F* ^2^ > 2σ(*F* ^2^)] = 0.045
*wR*(*F* ^2^) = 0.105
*S* = 0.95
5775 reflections
389 parameters
2 restraints
H atoms treated by a mixture of independent and constrained refinement
Δρ_max_ = 0.53 e Å^−3^
Δρ_min_ = −0.59 e Å^−3^

### 

Data collection: *APEX2* (Bruker, 2007 ▶); cell refinement: *SAINT* (Bruker, 2007 ▶); data reduction: *SAINT*; program(s) used to solve structure: *SHELXS97* (Sheldrick, 2008 ▶); program(s) used to refine structure: *SHELXL97* (Sheldrick, 2008 ▶); molecular graphics: *SHELXTL* (Sheldrick, 2008 ▶); software used to prepare material for publication: *SHELXTL*.

## Supplementary Material

Crystal structure: contains datablock(s) global, I. DOI: 10.1107/S1600536812009786/lh5429sup1.cif

Structure factors: contains datablock(s) I. DOI: 10.1107/S1600536812009786/lh5429Isup2.hkl

Supplementary material file. DOI: 10.1107/S1600536812009786/lh5429Isup3.cml

Additional supplementary materials:  crystallographic information; 3D view; checkCIF report

## Figures and Tables

**Table 1:** Hydrogen-bond geometry (Å, °)

*D*—H⋯*A*	*D*—H	H⋯*A*	*D*⋯*A*	*D*—H⋯*A*
N4—H4⋯O2^i^	0.90 (1)	1.95 (2)	2.827 (5)	166 (4)
N2—H2⋯O4	0.90 (1)	2.08 (2)	2.941 (5)	160 (4)
O3—H3⋯N3	0.82	1.87	2.569 (5)	143
O1—H1⋯N1	0.82	1.87	2.590 (5)	146

Symmetry code: (i) 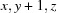.

**Fig. 1.**
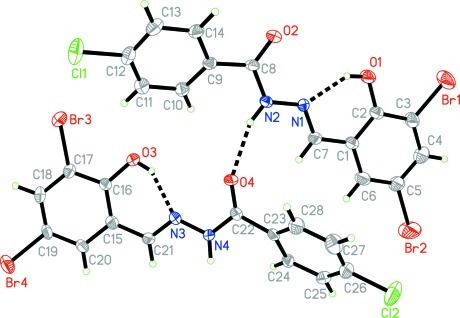
The asymmetric unit of the title compound, showing the atom-numbering scheme and displacement ellipsoids drawn at the 30% probability level. Hydrogen bonds are shown as dashed lines.

**Fig. 2.**
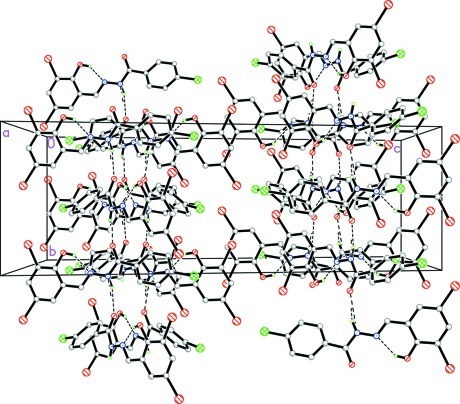
The molecular packing of the title compound viewed approximately along the a axis. Hydrogen bonds are shown as dashed lines. H-atoms not involved in hydrogen bonding have been omitted for clarity.

## supplementary crystallographic information

### Comment

Benzoylhydrazones are a kind of special Schiff bases bearing the –C(O)—NH—N═CH– group. Hydrazone compounds have received much attention for their excellent biological properties (Ajani *et al.*, 2010; Angelusiu *et al.*, 2010; Zhang *et al.*, 2010; Horiuchi *et al.*, 2009) as well as their crystal structures (Ban, 2010; Hussain *et al.*, 2010; Shalash *et al.*, 2010; Khaledi *et al.*, 2009). Recently, the author has reported some hydrazone compounds (Zhang, 2011; Zhang, 2012). In the present paper, the title new hydrazone compound, derived from the reaction of 3,5-dibromo-4-chlorobenzaldehyde with 4-chlorobenzohydrazide, is reported.

The asymmetric unit of the title hydrazone compound contains two independent molecules, as shown in Fig. 1. Each of the molecules of the compound adopts an *E* configuration about the C═N double bond. The dihedral angles between the two substituted benzene rings are 30.0 (2)° [C1-C6/C9-C14] and 51.6 (2)° [C15-C20/C23-C28]. In the crystal, molecules are linked through intermolecular N—H···O hydrogen bonds (Table 1), forming chains along the *b* axis (Fig. 2). In addition, there is an intramolecular O—H···N hydrogen bond in each molecule.

### Experimental

3,5-Dibromo-2-hydroxybenzaldehyde (0.280 g, 1 mmol) and 4-chlorobenzohydrazide (0.171 g, 1 mmol) were mixed in 50 ml methanol. The mixture was stirred and refluxed for 30 min and cooled to room temperature to give a colorless solution. Colorless block-shaped single crystals were obtained on slow evaporation of the solution in air.

### Refinement

H2 and H4 were located in a difference Fourier map and refined with the N—H distances restrained to 0.90 (1) Å. The remaining H atoms were positioned geometrically, with C—H = 0.93 Å, O—H = 0.82 Å, and with *U*_iso_(H) = 1.2*U*_eq_(C) and 1.5*U*_eq_(O).

### Crystal data

**Table:**

C_14_H_9_Br_2_ClN_2_O_2_	*F*(000) = 3360
*M**_r_* = 432.50	*D*_x_ = 1.850 Mg m^−^^3^
Monoclinic, *C*2/*c*	Mo *K*α radiation, λ = 0.71073 Å
Hall symbol: -C 2yc	Cell parameters from 2652 reflections
*a* = 21.0503 (19) Å	θ = 2.3–24.3°
*b* = 9.9895 (11) Å	µ = 5.40 mm^−^^1^
*c* = 30.185 (2) Å	*T* = 298 K
β = 101.836 (2)°	Block, colorless
*V* = 6212.4 (10) Å^3^	0.20 × 0.18 × 0.17 mm
*Z* = 16	

### Data collection

**Table:**

Bruker APEXII CCD area-detector diffractometer	5775 independent reflections
Radiation source: fine-focus sealed tube	2828 reflections with *I* > 2σ(*I*)
Graphite monochromator	*R*_int_ = 0.093
ω scans	θ_max_ = 25.5°, θ_min_ = 2.2°
Absorption correction: multi-scan (*SADABS*; Sheldrick, 1996)	*h* = −25→25
*T*_min_ = 0.412, *T*_max_ = 0.461	*k* = −12→12
21727 measured reflections	*l* = −36→33

### Refinement

**Table:**

Refinement on *F*^2^	Primary atom site location: structure-invariant direct methods
Least-squares matrix: full	Secondary atom site location: difference Fourier map
*R*[*F*^2^ > 2σ(*F*^2^)] = 0.045	Hydrogen site location: inferred from neighbouring sites
*wR*(*F*^2^) = 0.105	H atoms treated by a mixture of independent and constrained refinement
*S* = 0.95	*w* = 1/[σ^2^(*F*_o_^2^) + (0.0375*P*)^2^] where *P* = (*F*_o_^2^ + 2*F*_c_^2^)/3
5775 reflections	(Δ/σ)_max_ = 0.001
389 parameters	Δρ_max_ = 0.53 e Å^−^^3^
2 restraints	Δρ_min_ = −0.59 e Å^−^^3^

### Special details

**Table:**

Geometry. All e.s.d.'s (except the e.s.d. in the dihedral angle between two l.s. planes) are estimated using the full covariance matrix. The cell e.s.d.'s are taken into account individually in the estimation of e.s.d.'s in distances, angles and torsion angles; correlations between e.s.d.'s in cell parameters are only used when they are defined by crystal symmetry. An approximate (isotropic) treatment of cell e.s.d.'s is used for estimating e.s.d.'s involving l.s. planes.
Refinement. Refinement of *F*^2^ against ALL reflections. The weighted *R*-factor *wR* and goodness of fit *S* are based on *F*^2^, conventional *R*-factors *R* are based on *F*, with *F* set to zero for negative *F*^2^. The threshold expression of *F*^2^ > σ(*F*^2^) is used only for calculating *R*-factors(gt) *etc*. and is not relevant to the choice of reflections for refinement. *R*-factors based on *F*^2^ are statistically about twice as large as those based on *F*, and *R*- factors based on ALL data will be even larger.

### Fractional atomic coordinates and isotropic or equivalent isotropic displacement parameters (Å^2^)

**Table:**

	*x*	*y*	*z*	*U*_iso_*/*U*_eq_	
Br1	0.59984 (4)	−0.15649 (7)	0.50684 (2)	0.0893 (3)	
Br2	0.60096 (5)	0.40530 (8)	0.51878 (3)	0.1266 (4)	
Br3	0.16100 (3)	0.32156 (6)	0.13346 (2)	0.0621 (2)	
Br4	0.13920 (3)	0.88180 (6)	0.11656 (2)	0.0662 (2)	
Cl1	0.28553 (8)	0.0539 (2)	0.08490 (5)	0.0867 (6)	
Cl2	0.69898 (8)	0.5736 (2)	0.42951 (6)	0.0988 (7)	
N1	0.48103 (18)	0.0501 (4)	0.34395 (14)	0.0364 (10)	
N2	0.4503 (2)	0.0679 (4)	0.29989 (14)	0.0393 (11)	
N3	0.3518 (2)	0.5527 (4)	0.25814 (14)	0.0429 (11)	
N4	0.4089 (2)	0.5744 (4)	0.28790 (15)	0.0452 (12)	
O1	0.5259 (2)	−0.0988 (3)	0.41356 (11)	0.0564 (11)	
H1	0.5103	−0.0831	0.3869	0.085*	
O2	0.44485 (17)	−0.1529 (3)	0.28785 (12)	0.0522 (10)	
O3	0.26677 (17)	0.3947 (3)	0.21220 (12)	0.0469 (9)	
H3	0.3028	0.4132	0.2271	0.070*	
O4	0.43685 (17)	0.3578 (3)	0.28369 (11)	0.0496 (10)	
C1	0.5296 (2)	0.1408 (5)	0.41481 (18)	0.0409 (14)	
C2	0.5433 (2)	0.0165 (5)	0.43546 (18)	0.0444 (14)	
C3	0.5777 (3)	0.0117 (6)	0.48000 (19)	0.0544 (16)	
C4	0.5949 (3)	0.1262 (6)	0.50448 (19)	0.0688 (19)	
H4A	0.6173	0.1215	0.5344	0.083*	
C5	0.5787 (3)	0.2482 (6)	0.4843 (2)	0.0661 (19)	
C6	0.5472 (3)	0.2557 (5)	0.44034 (19)	0.0547 (16)	
H6	0.5373	0.3392	0.4271	0.066*	
C7	0.4980 (2)	0.1532 (5)	0.36838 (18)	0.0430 (14)	
H7	0.4896	0.2380	0.3558	0.052*	
C8	0.4314 (2)	−0.0407 (5)	0.27356 (18)	0.0385 (13)	
C9	0.3945 (2)	−0.0126 (5)	0.22721 (17)	0.0355 (13)	
C10	0.3549 (2)	0.0978 (5)	0.21643 (18)	0.0464 (15)	
H10	0.3515	0.1596	0.2389	0.056*	
C11	0.3200 (3)	0.1184 (6)	0.1727 (2)	0.0550 (16)	
H11	0.2930	0.1924	0.1657	0.066*	
C12	0.3265 (3)	0.0269 (6)	0.13990 (18)	0.0499 (15)	
C13	0.3631 (3)	−0.0858 (6)	0.1497 (2)	0.0564 (16)	
H13	0.3650	−0.1487	0.1273	0.068*	
C14	0.3976 (2)	−0.1056 (5)	0.19342 (19)	0.0460 (14)	
H14	0.4231	−0.1818	0.2003	0.055*	
C15	0.2601 (2)	0.6341 (5)	0.20665 (17)	0.0387 (13)	
C16	0.2386 (2)	0.5064 (5)	0.19194 (17)	0.0373 (13)	
C17	0.1875 (2)	0.4942 (5)	0.15544 (17)	0.0393 (13)	
C18	0.1587 (2)	0.6050 (6)	0.13239 (17)	0.0460 (14)	
H18	0.1263	0.5948	0.1066	0.055*	
C19	0.1784 (3)	0.7297 (5)	0.14789 (17)	0.0421 (14)	
C20	0.2292 (2)	0.7460 (5)	0.18405 (16)	0.0412 (14)	
H20	0.2431	0.8316	0.1935	0.049*	
C21	0.3177 (2)	0.6530 (5)	0.24191 (16)	0.0417 (14)	
H21	0.3297	0.7386	0.2526	0.050*	
C22	0.4500 (2)	0.4694 (5)	0.29903 (17)	0.0400 (14)	
C23	0.5113 (3)	0.5008 (5)	0.33136 (16)	0.0351 (13)	
C24	0.5150 (3)	0.5908 (5)	0.36667 (17)	0.0451 (15)	
H24	0.4781	0.6385	0.3696	0.054*	
C25	0.5719 (3)	0.6111 (5)	0.39751 (19)	0.0507 (15)	
H25	0.5731	0.6693	0.4217	0.061*	
C26	0.6262 (3)	0.5457 (6)	0.3924 (2)	0.0537 (16)	
C27	0.6246 (3)	0.4561 (6)	0.3576 (2)	0.0648 (18)	
H27	0.6620	0.4107	0.3545	0.078*	
C28	0.5668 (3)	0.4343 (5)	0.32730 (18)	0.0510 (15)	
H28	0.5656	0.3736	0.3038	0.061*	
H2	0.440 (2)	0.150 (2)	0.2886 (14)	0.039 (15)*	
H4	0.4239 (18)	0.6586 (17)	0.2928 (13)	0.028 (13)*	

### Atomic displacement parameters (Å^2^)

**Table:**

	*U*^11^	*U*^22^	*U*^33^	*U*^12^	*U*^13^	*U*^23^
Br1	0.1524 (8)	0.0620 (5)	0.0432 (4)	0.0235 (5)	−0.0042 (4)	0.0114 (4)
Br2	0.2209 (11)	0.0680 (5)	0.0661 (6)	−0.0248 (6)	−0.0282 (6)	−0.0228 (4)
Br3	0.0642 (4)	0.0549 (4)	0.0575 (4)	−0.0059 (3)	−0.0101 (3)	−0.0099 (3)
Br4	0.0726 (5)	0.0615 (4)	0.0599 (4)	0.0200 (3)	0.0025 (3)	0.0202 (4)
Cl1	0.0802 (12)	0.1304 (16)	0.0417 (10)	−0.0018 (12)	−0.0056 (9)	0.0045 (10)
Cl2	0.0554 (11)	0.1279 (17)	0.0980 (15)	−0.0097 (11)	−0.0196 (10)	−0.0172 (13)
N1	0.043 (3)	0.033 (3)	0.029 (3)	−0.004 (2)	−0.002 (2)	0.005 (2)
N2	0.054 (3)	0.025 (3)	0.035 (3)	0.003 (2)	0.000 (2)	0.000 (2)
N3	0.051 (3)	0.032 (3)	0.040 (3)	0.001 (2)	−0.004 (2)	0.005 (2)
N4	0.048 (3)	0.026 (3)	0.050 (3)	−0.004 (2)	−0.016 (2)	0.003 (2)
O1	0.093 (3)	0.035 (2)	0.035 (2)	0.003 (2)	−0.001 (2)	0.0007 (18)
O2	0.065 (3)	0.028 (2)	0.058 (3)	−0.0016 (19)	−0.002 (2)	0.0050 (19)
O3	0.054 (3)	0.034 (2)	0.046 (3)	−0.0019 (18)	−0.0069 (19)	−0.0033 (18)
O4	0.072 (3)	0.024 (2)	0.045 (2)	−0.0014 (18)	−0.009 (2)	−0.0068 (17)
C1	0.047 (3)	0.032 (3)	0.042 (4)	0.002 (3)	0.007 (3)	0.001 (3)
C2	0.052 (4)	0.033 (3)	0.047 (4)	0.003 (3)	0.006 (3)	0.000 (3)
C3	0.074 (4)	0.050 (4)	0.036 (4)	0.004 (3)	0.002 (3)	0.009 (3)
C4	0.097 (5)	0.069 (5)	0.030 (4)	−0.006 (4)	−0.010 (3)	−0.010 (4)
C5	0.099 (5)	0.051 (4)	0.038 (4)	−0.007 (4)	−0.011 (4)	−0.007 (3)
C6	0.079 (5)	0.038 (4)	0.045 (4)	−0.004 (3)	0.006 (3)	0.003 (3)
C7	0.054 (4)	0.033 (3)	0.040 (4)	−0.008 (3)	0.003 (3)	0.005 (3)
C8	0.040 (3)	0.023 (3)	0.050 (4)	−0.001 (3)	0.005 (3)	−0.001 (3)
C9	0.029 (3)	0.032 (3)	0.046 (4)	−0.003 (3)	0.007 (3)	−0.002 (3)
C10	0.053 (4)	0.041 (3)	0.042 (4)	0.007 (3)	0.000 (3)	−0.006 (3)
C11	0.057 (4)	0.049 (4)	0.054 (4)	0.006 (3)	0.000 (3)	0.001 (3)
C12	0.044 (4)	0.069 (4)	0.035 (4)	−0.011 (3)	0.003 (3)	0.001 (3)
C13	0.058 (4)	0.059 (4)	0.048 (4)	−0.007 (3)	0.004 (3)	−0.016 (3)
C14	0.044 (4)	0.038 (3)	0.052 (4)	0.005 (3)	0.001 (3)	−0.012 (3)
C15	0.043 (3)	0.039 (3)	0.035 (3)	0.003 (3)	0.009 (3)	0.003 (3)
C16	0.043 (3)	0.030 (3)	0.037 (3)	0.004 (3)	0.004 (3)	0.001 (3)
C17	0.037 (3)	0.046 (3)	0.034 (3)	0.003 (3)	0.004 (3)	−0.005 (3)
C18	0.045 (4)	0.062 (4)	0.028 (3)	0.008 (3)	0.001 (3)	0.004 (3)
C19	0.051 (4)	0.044 (4)	0.029 (3)	0.010 (3)	0.003 (3)	0.010 (3)
C20	0.049 (4)	0.033 (3)	0.041 (4)	0.005 (3)	0.009 (3)	0.009 (3)
C21	0.055 (4)	0.033 (3)	0.035 (3)	−0.008 (3)	0.003 (3)	0.003 (3)
C22	0.049 (4)	0.034 (3)	0.033 (3)	−0.002 (3)	0.000 (3)	0.006 (3)
C23	0.047 (4)	0.027 (3)	0.029 (3)	0.002 (3)	0.003 (3)	0.006 (2)
C24	0.052 (4)	0.031 (3)	0.045 (4)	0.010 (3)	−0.009 (3)	−0.006 (3)
C25	0.057 (4)	0.037 (3)	0.053 (4)	−0.007 (3)	0.000 (3)	−0.005 (3)
C26	0.042 (4)	0.056 (4)	0.058 (4)	−0.005 (3)	−0.001 (3)	0.005 (3)
C27	0.055 (4)	0.071 (5)	0.071 (5)	0.015 (4)	0.020 (4)	0.003 (4)
C28	0.058 (4)	0.054 (4)	0.042 (4)	−0.001 (3)	0.010 (3)	−0.003 (3)

### Geometric parameters (Å, º)

**Table:**

Br1—C3	1.882 (5)	C9—C10	1.380 (6)
Br2—C5	1.888 (6)	C9—C14	1.391 (6)
Br3—C17	1.890 (5)	C10—C11	1.389 (7)
Br4—C19	1.888 (5)	C10—H10	0.9300
Cl1—C12	1.727 (5)	C11—C12	1.374 (7)
Cl2—C26	1.724 (6)	C11—H11	0.9300
N1—C7	1.274 (5)	C12—C13	1.363 (7)
N1—N2	1.366 (5)	C13—C14	1.383 (7)
N2—C8	1.356 (6)	C13—H13	0.9300
N2—H2	0.897 (10)	C14—H14	0.9300
N3—C21	1.271 (5)	C15—C16	1.396 (6)
N3—N4	1.362 (5)	C15—C20	1.398 (6)
N4—C22	1.357 (6)	C15—C21	1.452 (7)
N4—H4	0.900 (10)	C16—C17	1.379 (6)
O1—C2	1.341 (5)	C17—C18	1.379 (6)
O1—H1	0.8200	C18—C19	1.364 (7)
O2—C8	1.213 (5)	C18—H18	0.9300
O3—C16	1.349 (5)	C19—C20	1.373 (7)
O3—H3	0.8200	C20—H20	0.9300
O4—C22	1.217 (5)	C21—H21	0.9300
C1—C6	1.390 (6)	C22—C23	1.484 (6)
C1—C2	1.393 (6)	C23—C28	1.371 (7)
C1—C7	1.428 (7)	C23—C24	1.384 (6)
C2—C3	1.390 (7)	C24—C25	1.374 (7)
C3—C4	1.370 (7)	C24—H24	0.9300
C4—C5	1.374 (7)	C25—C26	1.352 (7)
C4—H4A	0.9300	C25—H25	0.9300
C5—C6	1.357 (7)	C26—C27	1.376 (7)
C6—H6	0.9300	C27—C28	1.380 (7)
C7—H7	0.9300	C27—H27	0.9300
C8—C9	1.482 (6)	C28—H28	0.9300
			
C7—N1—N2	118.6 (4)	C12—C13—H13	120.4
C8—N2—N1	119.3 (4)	C14—C13—H13	120.4
C8—N2—H2	119 (3)	C13—C14—C9	120.6 (5)
N1—N2—H2	121 (3)	C13—C14—H14	119.7
C21—N3—N4	118.8 (4)	C9—C14—H14	119.7
C22—N4—N3	118.2 (4)	C16—C15—C20	119.2 (5)
C22—N4—H4	120 (3)	C16—C15—C21	121.3 (5)
N3—N4—H4	119 (3)	C20—C15—C21	119.3 (5)
C2—O1—H1	109.5	O3—C16—C17	119.2 (5)
C16—O3—H3	109.5	O3—C16—C15	121.9 (4)
C6—C1—C2	118.7 (5)	C17—C16—C15	119.0 (5)
C6—C1—C7	119.3 (5)	C18—C17—C16	121.5 (5)
C2—C1—C7	121.9 (5)	C18—C17—Br3	119.2 (4)
O1—C2—C3	118.8 (5)	C16—C17—Br3	119.1 (4)
O1—C2—C1	122.3 (5)	C19—C18—C17	119.3 (5)
C3—C2—C1	118.9 (5)	C19—C18—H18	120.4
C4—C3—C2	121.3 (5)	C17—C18—H18	120.4
C4—C3—Br1	119.9 (4)	C18—C19—C20	120.9 (5)
C2—C3—Br1	118.8 (4)	C18—C19—Br4	119.5 (4)
C3—C4—C5	119.1 (5)	C20—C19—Br4	119.5 (4)
C3—C4—H4A	120.4	C19—C20—C15	120.1 (5)
C5—C4—H4A	120.4	C19—C20—H20	119.9
C6—C5—C4	120.6 (5)	C15—C20—H20	119.9
C6—C5—Br2	120.6 (5)	N3—C21—C15	120.1 (5)
C4—C5—Br2	118.8 (4)	N3—C21—H21	120.0
C5—C6—C1	121.2 (5)	C15—C21—H21	120.0
C5—C6—H6	119.4	O4—C22—N4	122.0 (5)
C1—C6—H6	119.4	O4—C22—C23	122.9 (5)
N1—C7—C1	121.1 (5)	N4—C22—C23	115.1 (5)
N1—C7—H7	119.5	C28—C23—C24	118.0 (5)
C1—C7—H7	119.5	C28—C23—C22	118.8 (5)
O2—C8—N2	120.8 (5)	C24—C23—C22	123.2 (5)
O2—C8—C9	123.4 (5)	C25—C24—C23	121.3 (5)
N2—C8—C9	115.8 (4)	C25—C24—H24	119.3
C10—C9—C14	118.6 (5)	C23—C24—H24	119.3
C10—C9—C8	123.3 (5)	C26—C25—C24	119.5 (5)
C14—C9—C8	118.1 (5)	C26—C25—H25	120.2
C9—C10—C11	121.2 (5)	C24—C25—H25	120.2
C9—C10—H10	119.4	C25—C26—C27	120.8 (5)
C11—C10—H10	119.4	C25—C26—Cl2	120.5 (5)
C12—C11—C10	118.4 (5)	C27—C26—Cl2	118.8 (5)
C12—C11—H11	120.8	C26—C27—C28	119.3 (6)
C10—C11—H11	120.8	C26—C27—H27	120.3
C13—C12—C11	121.9 (5)	C28—C27—H27	120.3
C13—C12—Cl1	119.1 (5)	C23—C28—C27	121.0 (5)
C11—C12—Cl1	119.0 (5)	C23—C28—H28	119.5
C12—C13—C14	119.3 (5)	C27—C28—H28	119.5

### Hydrogen-bond geometry (Å, º)

**Table:**

*D*—H···*A*	*D*—H	H···*A*	*D*···*A*	*D*—H···*A*
N4—H4···O2^i^	0.90 (1)	1.95 (2)	2.827 (5)	166 (4)
N2—H2···O4	0.90 (1)	2.08 (2)	2.941 (5)	160 (4)
O3—H3···N3	0.82	1.87	2.569 (5)	143
O1—H1···N1	0.82	1.87	2.590 (5)	146

Symmetry code: (i) *x*, *y*+1, *z*.

## References

[bb1] Ajani, O. O., Obafemi, C. A., Nwinyi, O. C. & Akinpelu, D. A. (2010). *Bioorg. Med. Chem.* **18**, 214–221. 10.1016/j.bmc.2009.10.064 19948407

[bb2] Angelusiu, M. V., Barbuceanu, S. F., Draghici, C. & Almajan, G. L. (2010). *Eur. J. Med. Chem.* **45**, 2055–2062. 10.1016/j.ejmech.2010.01.033 20133023

[bb3] Ban, H.-Y. (2010). *Acta Cryst.* E**66**, o3240. 10.1107/S160053681004701X PMC3011623 21589529

[bb4] Bruker (2007). *APEX2* and *SAINT* Bruker AXS Inc., Madison, Wisconsin, USA.

[bb5] Horiuchi, T., Nagata, M., Kitagawa, M., Akahane, K. & Uoto, K. (2009). *Bioorg. Med. Chem.* **17**, 7850–7860. 10.1016/j.bmc.2009.10.039 19889545

[bb6] Hussain, A., Shafiq, Z., Tahir, M. N. & Yaqub, M. (2010). *Acta Cryst.* E**66**, o1888. 10.1107/S1600536810025390 PMC3007219 21588224

[bb7] Khaledi, H., Saharin, S. M., Mohd Ali, H., Robinson, W. T. & Abdulla, M. A. (2009). *Acta Cryst.* E**65**, o1920. 10.1107/S1600536809027032 PMC2977291 21583605

[bb8] Shalash, M., Salhin, A., Adnan, R., Yeap, C. S. & Fun, H.-K. (2010). *Acta Cryst.* E**66**, o3126–o3127. 10.1107/S1600536810045162 PMC3011781 21589430

[bb9] Sheldrick, G. M. (1996). *SADABS* University of Göttingen, Germany.

[bb10] Sheldrick, G. M. (2008). *Acta Cryst.* A**64**, 112–122. 10.1107/S0108767307043930 18156677

[bb11] Zhang, W.-G. (2011). *Acta Cryst.* E**67**, o233.

[bb12] Zhang, W.-G. (2012). *Acta Cryst.* E**68**, o357. 10.1107/S1600536812000463 PMC3275039 22346984

[bb13] Zhang, Y. H., Zhang, L., Liu, L., Guo, J. X., Wu, D. L., Xu, G. C., Wang, X. H. & Jia, D. Z. (2010). *Inorg. Chim. Acta*, **363**, 289–293.

